# Favorable Outcome of COVID-19 Pneumonia in a Kidney Transplant Recipient Treated with Tocilizumab

**DOI:** 10.1155/2020/8830626

**Published:** 2020-09-16

**Authors:** Adnane Guella, Osman Elfadil, Ghisson Abdulrazaq, Sahla Osman, Mohammed Munir Khan, Abdalla Ahmed, Feras Deyab

**Affiliations:** ^1^Nephrology Section, Sharjah University Hospital, Sharjah, UAE; ^2^Department of Internal Medicine, Sharjah University Hospital, Sharjah, UAE

## Abstract

The presentation of COVID-19 pneumonia in kidney transplant recipients is similar to that of the general population. However, in the former, it may have a worse clinical course. We report a kidney transplant patient affected by COVID-19 pneumonia whose condition worsened 9 days after the initial presentation. As no therapeutic guidelines on the subject are currently available, here we share our approach in the management of the immunosuppressive medications and the antiviral therapy and compare them to the scarce available data. We also expose the use of tocilizumab in our patient with excellent results.

## 1. Introduction

COVID-19 pneumonia in kidney transplant recipients represents a new challenging situation for nephrologists and physicians dealing with COVID infection. Because the disease is new, no therapeutic strategy is available so far, regarding either the current immunosuppressive medications of the patient or the antiviral therapy.

Data from a recent large series show that clinical, biological, and radiological presentations of COVID-19 pneumonia in kidney transplant recipients are similar to those of the general population although the course of the disease may be more severe [1, 2].

Here, we share our experience with the successful management of a kidney transplant recipient whose COVID-19 pneumonia worsened 9 days after the initial presentation. The use of a single intravenous shot of tocilizumab was followed by a remarkable improvement. Few recent papers showed a similar outcome, opening the door to ongoing randomized trials on tocilizumab in kidney transplant recipients [3].

## 2. Case Report

A 65-year-old male kidney transplant patient presented to the emergency department on 23/04/2020 complaining of fever, cough, soared throat, and diarrhea for three days. His temperature was 38.3; oxygen saturation was 94% in room air with 18 breaths per minute. Blood pressure was 130/70. In his history, we noticed a live unrelated kidney transplantation done in 1987 and diabetes mellitus for the last six years. His initial renal disease was unknown. His medications at that time included cyclosporine (75 mg twice daily) and Myfortic (720 mg twice daily). His creatinine was ranging between 210 and 240 for the last 4 years.

**Figure 1:**
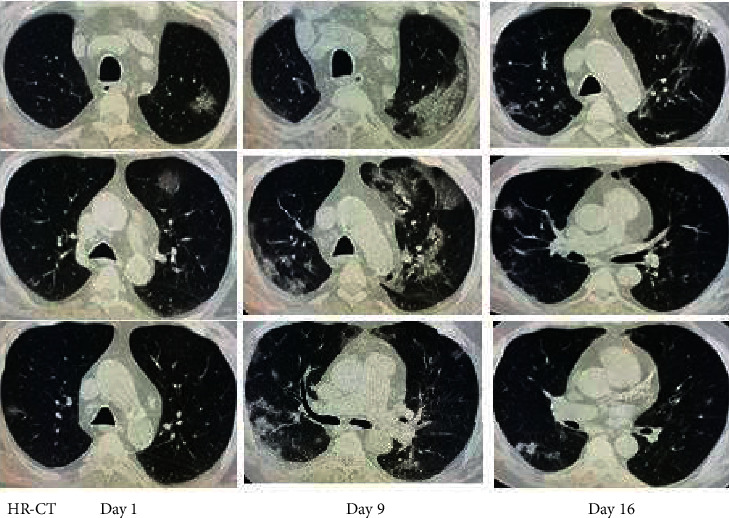
HR-CT findings on day 1 with few focal ground-glass opacifications (GGO), day 9 with GGO dissemination, and day 16 with substantial improvement of the lung parenchyma.

A chest X-ray showed left peripheral midzone opacity with bilateral exaggerated lung markings and peribronchial thickening suggesting bronchopneumonia. A nasopharyngeal COVID swab was done followed by an HR-CT of the chest which revealed bilateral round-shape ground-glass opacifications in both lungs showing peripheral distribution in favor of COVID-19 pneumonia (Figure 1, day 1). Blood investigations showed high blood cell count (12700 cells/mm³) with no lymphopenia, high CRP (126 mg/dl), high procalcitonin (PCT) (1.3 ng/ml), ferritin (1420 *μ*g/l), and slightly high LDH and D-dimer (223 U/l and 0.7 mg/l, respectively).

Therefore, the patient was admitted in the COVID isolation ward. He was started on ceftriaxone and hydroxychloroquine (400 mg twice the first day and 200 mg twice subsequently). QT interval was assessed regularly and was normal during the 10-day course of hydroxychloroquine. COVID-19 was detected by PCR. On admission, mycophenolate mofetil (MMF) was discontinued, and Clexane was prescribed at a dose of 40 mg daily. On day 4, as the patient remained highly febrile, ceftriaxone was replaced by meropenem. Blood investigations showed worsening of CRP (155), ferritin (6910), and D-dimer (2.2). Cyclosporine dose was reduced to 25 mg twice, and prednisolone 10 mg was introduced.

On day 9, the cough worsened with moderate tachypnea and persistent diarrhea and fever. Saturation of oxygen dropped to 91%. The patient developed leukopenia with lymphopenia (3.5 and 0.7, respectively) for the first time since admission. The inflammatory markers showed persistent increase (CRP 176 and PCT jumped to 84.2). A repeated HR-CT revealed worsening of chest findings (Figure 1, day 9). We therefore decided to give a single shot of tocilizumab 400 mg intravenously and start the patient on Kaletra (lopinavir/ritonavir) and prednisolone 10 mg daily (Figure 1).

Within the next two days, we noticed a dramatic improvement of his clinical condition with disappearance of fever and diarrhea and reduced intensity of the cough. The inflammatory markers also improved: CRP 45 and PCT 36. Leucocytes and lymphocytes increased (5.2 and 1.6, respectively). On day 16, HR-CT was repeated and showed almost complete clearance of the previous lesions (Figure 1, day 16). The patient was completely asymptomatic, and the inflammatory markers were all back to normal. At this time, cyclosporine dose was increased to 50 mg twice a day. MMF was resumed once PCR for COVID-19 came back negative (day 18). During the whole admission, blood sugar was well controlled, with glycated hemoglobin ranging between 6.5 and 7 (Table 1).

## 3. Discussion

The management of COVID-19 pneumonia in kidney transplant patients is a challenging situation as no optimal strategy is currently known. In practice, nephrologists face two issues: firstly, how to deal with the current immunosuppressive therapy of the transplant patients, and secondly, what antiviral therapy to be used. From the scarce information available in the literature, the general practice is that antimetabolites are withdrawn, calcineurin inhibitors (CNIs) are either held or used at reduced dose, and prednisolone is maintained at the same or higher dose, or it is introduced if patients were not on it previously [1, 2, 4–6]. In spite of these modifications, no acute rejection has been reported so far. The management of our patient conforms to this practice: i.e., discontinuing MMF, introducing prednisolone, and reducing cyclosporine dose to the minimum. Few authors postulated that the current practice with CNIs (i.e., held or used at reduced dose) not only exposes the patients to acute rejection [7] but also deprives them from drugs (cyclosporine and tacrolimus) that have been shown to inhibit viral replication of human coronaviruses in vitro [8, 9]. However, so far the role of CNIs in the viral replication of SARS-CoV-2 remains unknown, and therefore, the common practice with CNIs may prevail. In our patient, eGFR improved with the reduction of cyclosporine dose.

For the antiviral therapy, kidney transplant patients are treated in the same way as the nontransplant population, and the choice is made based on the severity of the condition [1, 4–6]. Wu et al. analyzed the risk factors associated with COVID-19 pneumonia and showed a significant increase of IL-6 [10]. Therefore, tocilizumab, a humanized monoclonal antibody that competitively inhibits IL‐6 by binding to its receptors, found logical off-label utilization. It is currently being assessed in an ongoing multicenter study in Italy [3]. So far, tocilizumab is used in moderate and severe cases of COVID-19 pneumonia where a hyperinflammatory state or cytokine-release syndrome is present [7, 11]. Data related to the use of tocilizumab are still limited but encouraging as no side effect has been reported so far [2, 12, 13].Our patient received tocilizumab as a single shot at the time where his cough and chest HR-CT worsened. Although blood level of IL 6 was not available for our patient, we considered the high level of PCT which has been associated in the series of Pereira et al. to the severity of the disease, another argument to start tocilizumab [12]. Our patient showed a remarkable clinical improvement within two days following the injection of tocilizumab. We consider this improvement clearly related to tocilizumab and not to hydroxychloroquine or Kaletra. Tocilizumab was used when the patient's condition worsened at the end of a 10-day course of hydroxychloroquine. On the contrary, Kaletra was started on the same day of the tocilizumab single shot, but it is highly unlikely to be responsible for this rapid clinical improvement as its treatment course is 10 days.

The prognosis of COVID-19 pneumonia in the renal transplant population is varying. In the series of Zhu et al. [2], 9 out of ten transplant patients recovered successfully although the severity of the pneumonia was greater than in the control group. In the series of Pereira et al., severe cases showed poorer outcomes compared to moderate cases [12]. Therefore, tocilizumab may be of great help in the transplant population. In conclusion, we presented in this paper our approach in managing COVID-19 pneumonia in a kidney transplant patient and showed the favorable impact of tocilizumab in the management.

## Figures and Tables

**Table 1:** Clinical and laboratory data before and after tocilizumab injection.

Days	D1	D4	D9	D10	D12	D14	D16
Clinically	Fever	Fever	Fever+^*∗*^	Fever+^*∗*^	^*∗∗*^	^*∗∗∗*^	^*∗∗∗*^
WBC Lymphocytes	12,700 2.5%	9600 1.9%	3500 0.7%		5.2 1.6%	5.8 2.1%	10.4 2.2%
CRP/procal	126/1.3	155/1.54	176/84.2		45/36	7.3/3.9	<2/0.81
Ferritin D-dimer	1420 0.7	6910 2.2	4633 1.2		3900 1.0	2105 0.8	1700 0.9
eGFR	21	24	24		31	38	36
Rx	Tocilizumab						
HR-CT	Done		Done				Done

^*∗*^Severe diarrhea, worsening of cough, tachypnea, and O_2_ desaturation. ^*∗∗*^No fever, no diarrhea, and cough less. ^*∗∗∗*^Asymptomatic.

## Data Availability

The data used to support the findings of this study are included within the article.
